# Whole-Genome Sequencing to Detect Numerous *Campylobacter jejuni* Outbreaks and Match Patient Isolates to Sources, Denmark, 2015–2017 

**DOI:** 10.3201/eid2603.190947

**Published:** 2020-03

**Authors:** Katrine G. Joensen, Kristoffer Kiil, Mette R. Gantzhorn, Birgitte Nauerby, Jørgen Engberg, Hanne M. Holt, Hans L. Nielsen, Andreas M. Petersen, Katrin G. Kuhn, Gudrun Sandø, Steen Ethelberg, Eva M. Nielsen

**Affiliations:** Statens Serum Institut, Copenhagen, Denmark (K.G. Joensen, K. Kiil, K.G. Kuhn, S. Ethelberg, E.M. Nielsen);; Danish Veterinary and Food Administration, Copenhagen (M.R. Gantzhorn, B. Nauerby, G. Sandø);; Slagelse Hospital, Slagelse, Denmark (J. Engberg);; Odense University Hospital, Odense, Denmark (H.M. Holt);; Aalborg University Hospital, Aalborg, Denmark (H.L. Nielsen);; Aalborg University, Aalborg (H.L. Nielsen);; Hvidovre University Hospital, Hvidovre, Denmark (A.M. Petersen)

**Keywords:** Campylobacter, bacteria, whole-genome sequencing, epidemiology, outbreaks, core-genome multilocus sequence typing, cgMLST, foodborne infections, molecular subtyping, Denmark

## Abstract

In industrialized countries, the leading cause of bacterial gastroenteritis is *Campylobacter jejuni*. However, outbreaks are rarely reported, which may reflect limitations of surveillance, for which molecular typing is not routinely performed. To determine the frequency of genetic clusters among patients and to find links to concurrent isolates from poultry meat, broiler chickens, cattle, pigs, and dogs, we performed whole-genome sequencing on 1,509 *C. jejuni* isolates from 774 patients and 735 food or animal sources in Denmark during 2015–2017. We found numerous clusters; 366/774 (47.3%) clinical isolates formed 104 clusters of >2 isolates. A total of 41 patient clusters representing 199/366 (54%) patients matched a potential source, primarily domestic chickens/broilers. This study revealed serial outbreaks and numerous matches to concurrent food and animal isolates and highlighted the potential of whole-genome sequencing for improving routine surveillance of *C. jejuni* by enhancing outbreak detection, source tracing, and potentially prevention of human infections.

*Campylobacter jejuni* is the most frequent cause of bacterial gastroenteritis in industrialized countries worldwide (*1*,*2*), including in Denmark, where ≈4,000 *Campylobacter* infections are reported annually (*3*). Despite the high notification rates, *Campylobacter* infections are believed to be highly underdiagnosed (*4*,*5*). Denmark’s national surveillance system for *Campylobacter* is based on observation of the number of notified human cases (*3*); a substantial number of *Campylobacter* outbreaks may be overlooked because of a lack of routine microbiological typing of isolates. This hypothesis is supported by evidence from a recent study, in which whole-genome sequencing (WGS)–based typing of selected clinical *Campylobacter* isolates from patients in Denmark identified numerous small outbreak-like clusters (*6*). This finding suggests that more outbreaks occur in Denmark than the few typically large outbreaks associated with a single event that are detected by the current surveillance system (*7*–*10*). To achieve a national surveillance system that will detect ongoing *Campylobacter* outbreaks in real time, highly discriminatory subtyping of isolates is needed. Thus, we more comprehensively evaluated the frequency of *Campylobacter* outbreaks among humans in Denmark by using WGS-based typing. To match the clinical isolates with their sources, we compared them with isolates from food and animals, covering the main putative sources of human *Campylobacter* infections (i.e., contact with or consumption of animals or animal products, primarily contaminated poultry meat). Although *Campylobacter* infections are primarily foodborne, a recent case–control study in Denmark found that contact with animals and the environment might account for a substantial proportion of domestic infections (*11*). Several other reported sources of infection include unpasteurized milk, drinking water, bathing water, vegetables, and fruits (*1*,*12*–*14*).

WGS offers high-resolution discriminatory subtyping and has been successfully implemented for public health surveillance of several foodborne pathogens (e.g., *Salmonella*, *Listeria,* and Shiga toxin–producing *Escherichia coli*) in Denmark and abroad (*3*,*15*,*16*). Recent studies have proven WGS to be applicable for *Campylobacter* outbreak investigations (*17*–*22*), and it has recently been shown that WGS could trace back clinical infections directly to chicken slaughter batches (*23*).

For this study, we performed WGS on a large cohort of 1,509 *C. jejuni* isolates collected from patients and food/animal samples in Denmark over 2 years. We clustered the isolates by using core-genome multilocus sequence typing (cgMLST) to determine the frequency of genetic clusters among clinical isolates (i.e., possible outbreaks) and how they matched concurrent isolates from potential sources. We detected numerous clusters among the patients and a large number of matches between clinical isolates and potential food/animal sources.

## Materials and Methods

### Isolate Collection

During 2015–2017, we collected *Campylobacter* isolates representing human infections and food/animal sources. The collection period for isolates from humans (clinical isolates) and from food/animals overlapped by 18 months (October 2015–March 2017). We present WGS data for 1,509 *C. jejuni* isolates: 774 clinical and 735 food/animal.

The clinical isolates were supplied by 4 clinical microbiological laboratories (Aalborg University Hospital, Slagelse Hospital, Hvidovre University Hospital, and Odense University Hospital), which were performing clinical microbiological services for the regional hospitals and practitioners in 4 of the 5 geographic regions in Denmark. These 4 laboratories diagnosed ≈60% of the *Campylobacter* cases in Denmark. Collection of 904 isolates was timed to best capture seasonal variation. On the basis of data from previous years, each laboratory was to supply the number of isolates that could be expected to constitute a constant fraction of their positive samples per calendar month.

We performed WGS on all collected isolates. After removing 130 sequences because of poor sequence quality, we included in our analysis 774 *C. jejuni* isolates (representing 772 patients), representing ≈10% of the *Campylobacter* cases during the period.

Clinical isolates were collected during September 2015–April 2017 and represented 673 domestically acquired infections, 60 travel-associated infections, and 41 infections in persons of unknown travel status. We also included 14 isolates obtained from a fifth region (Aarhus University Hospital) during an outbreak investigation in June 2017. Of the domestically acquired isolates, 192 had been previously analyzed as part of another study to detect clusters among *Campylobacter* isolates in Denmark (October 2015–June 2016) (*6*).

The *C. jejuni* isolates from food/animals were collected during May 2015–March 2017 by the Danish Veterinary and Food Administration, where a total of 798 food/animal isolates were subjected to WGS. A total of 63 isolates were removed from the study because of poor sequence quality. The remaining 735 isolates represented animals from Denmark, hereafter referred to as domestic (27 pigs, 214 cattle, and 150 broilers [collected as cecal samples at slaughter]) or retail meat (172 domestic chicken, 111 imported chicken, 9 imported turkey, 4 domestic duck, and 22 imported duck). A limited number of isolates were available from 3 domestic retail vegetables, 2 seawater samples, and 21 pet dogs. The food/animal samples were primarily collected as part of official surveillance programs. Some animals, as well as turkey meat and duck meat, were collected specifically for the source attribution study (*24*). Isolates from pet dogs were obtained from healthy dogs and dogs that had diarrhea before sampling.

### WGS

Genomic DNA (gDNA) from clinical isolates was purified by using a QIAGEN DNeasy Blood and Tissue kit (https://www.qiagen.com), and gDNA from food/animal isolates was purified by using the Invitrogen Easy-DNA gDNA purification kit (https://www.thermofisher.com). Sequencing was conducted by using Illumina technology (https://www.illumina.com) on either MiSeq or NextSeq sequencing machines and using the Nextera XT Library preparation protocol for paired-end reads of 150 bp or 250 bp. Raw data were deposited in the European Nucleotide Archive (accession no. PRJEB31119).

We processed WGS data by using a QC-pipeline (https://github.com/ssi-dk/SerumQC), in which isolate sequences were removed in case of contamination with >5% of another genus, as well as sequences representing *C. jejuni* isolates with genome sizes outside the range of 1.6–1.9 Mbp. We conducted further analyses by using BioNumerics 7.6 (Applied Maths, https://www.applied-maths.com), in which cgMLST assignment was based on the 1,343-loci cgMLST scheme from the *Campylobacter* Multi Locus Sequence Typing website (https://pubmlst.org/campylobacter) at the University of Oxford (*25*). We removed isolate sequences from the dataset if assemblies comprised >500 contigs, the cgMLST core-percentage was <95%, or >40 loci were present with multiple consensus sequences. When multiple isolates were available from the same patient, we kept only unique representative sequences. Clusters, or matches, were defined by the unweighted pair group method with arithmetic mean (UPGMA) clustering with cgMLST allele differences as a distance measure and a cluster threshold of 4. This value was chosen after evaluation of the phylogeny, with clustering of potentially related isolates and separation of nonrelated isolates (Appendix). We gave each cluster a unique name in which sequence type (ST) was followed by a number (#1, #2, etc.) to separate different clusters of the same ST.

### Rarefaction

We produced a rarefaction curve for the clinical isolates by using the rarefy function from the R vegan library (*26*). For each isolate, we used the specific cluster type as the input type for the rarefaction and assigned sporadic cases their own cluster type. We calculated data points for a subsample of n_subsample_ = 100 to 774, with a step of 100. From a random reordering of the same samples, at the same subsample steps as above, we selected the first n_subsample_ samples and counted the number of clusters of size >1 and >4.

### Cost-effectiveness Calculation

Estimates of outbreak detection frequency were calculated by using the survival function S(cluster_threshold-1) under the binomial distribution B(N_cases_,f_sampling_). The complete code for the cost-effectiveness calculation is provided (Appendix).

## Results

**Figure 1:**
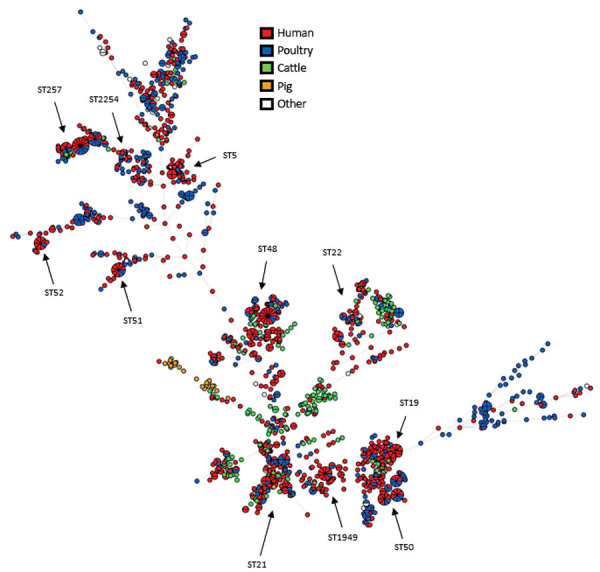
Overall phylogeny of 1,509 *Camplylobacter jejuni* isolates from Denmark, 2015–2017. Minimum spanning tree was based on core-genome multilocus sequence typing data. Colors indicate specific origin. Each small circle represents single isolates, and pie chart graphics represent multiple isolates with 0 allelic differences. Branch lengths represent the genetic (allelic) distances. The dataset displayed high diversity, and many genetic clusters were detected, in total 104 clusters of clinical isolates (n = 366). Forty-one of these clusters matched food/animal isolates; 34 matches to food/animal isolates were detected for sporadic clinical cases.

The 1,509 sequenced *C. jejuni* isolates showed high diversity and were distributed over 234 seven-locus multilocus sequence types (MLSTs) (Figure 1). Of the sources represented by numerous isolates, only the isolates from pigs (n = 27) were confined to a single clonal complex (CC), CC403, with the exception of 2 CC21 isolates. The clinical isolates and poultry isolates were generally distributed throughout the tree, although poultry isolates were not present in CC403. Isolates from cattle were abundant in certain clonal complexes (CC21, CC42, CC48, and CC61) and generally present in most parts of the tree. Despite the high genetic diversity of the dataset in general, we identified a large number of genetic clusters.

### Genetic Clusters

Analysis of cgMLST results for all 1,509 isolates indicated that 732 of the isolates were part of 204 clusters consisting of >2 isolates. Of these, 63 clusters consisted exclusively of clinical isolates (n = 167), 66 consisted exclusively of food/animal isolates (n = 176), and 75 consisted of clinical (n = 233) and food/animal isolates (n = 156).

### Clusters of Clinical Isolates

**Figure 2:**
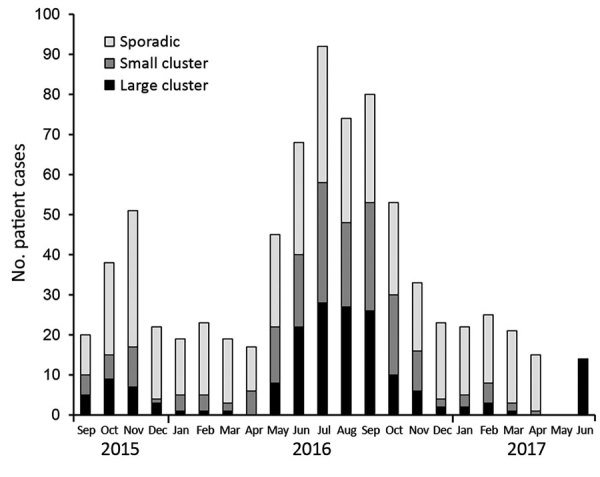
Distribution of clinical *Camplylobacter jejuni* isolates from Denmark over time, 2015–2017. Colors represent isolates in large (>5 isolates, n = 176) and small (2–4 isolates, n = 190) clusters or as sporadic cases (n = 408). All 774 clinical isolates are shown according to their sample date. A higher concentration of clusters occurred during the summer, and the number of sporadic cases was relatively constant during the year.

Separate analysis of the 774 clinical isolates showed that 366 (47.3%) formed 104 clusters consisting of >2 isolates. A considerably higher concentration of clusters occurred during the summer, when *Campylobacter* incidence peaks (*27*), whereas the number of sporadic infections was almost constant throughout the year (Figure 2).

Most (82) clusters were small clusters of 2–4 clinical isolates (total 190 isolates); 22 large clusters consisted of 5–17 clinical isolates (total 176 isolates). We detected a direct match to >1 isolates of food or animal origin for most (17/22; 77%) of the large patient clusters but only 24 (29%) of the 82 small patient clusters. Thus, 41/104 patient clusters could be matched to a potential source and represented 199/366 (54%) of the patients who were part of a potential outbreak.

**Figure 3:**
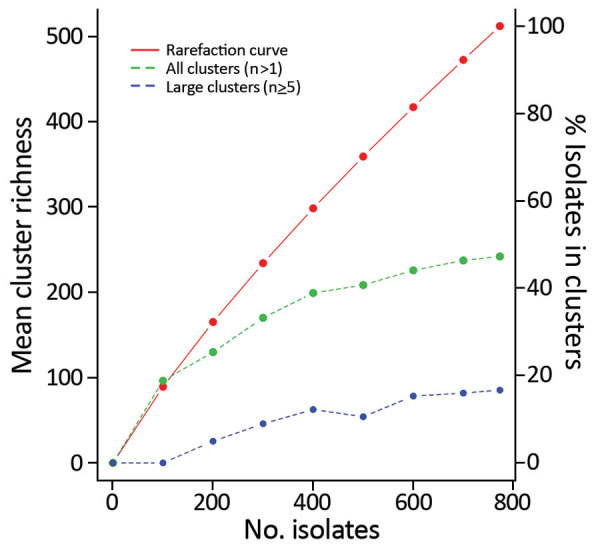
Rarefaction curve of *Campylobacter jejuni* clinical isolates from Denmark, 2015–2017. Curve of cluster types is shown relating to the left y-axis, revealing 512 different cluster types, which encompass the 104 defined clusters as well as the 408 sporadic cases assigned to their own cluster type. The right y-axis indicates the percentage of clinical isolates assigned to any cluster or a large cluster for a given subsample size. The rarefaction curve indicates that only a small fraction of the diversity is sampled; a larger fraction of the sporadic isolates would be included in clusters if a greater fraction of the population had been sampled.

The clinical isolates in this study represented ≈10% of the nationwide cases in the period. More extensive sampling would probably lead to detection of more clusters. Typing of all 774 clinical isolates indicated that 47% were part of the 104 identified clusters (Figure 3); had the sample size been reduced to, for example, 400 or 200 isolates, the percentage of isolates in clusters would have decreased to 39% or 25%, respectively.

To implement cost-effective surveillance of clusters of clinical isolates, it is essential to consistently find large clusters without investigating small insignificant clusters. With a sampling frequency of 10% and an investigation threshold of 4 clustered isolates, we expect to find 99.2% of large outbreaks (of 100 reported cases), and an investigation would be triggered within the first one third of the cases for 42% of the large outbreaks. A small outbreak of 10 reported cases would be observed only 1.3% of the time, and assuming a 1:100 ratio of large:small outbreaks, an average of 1.3 small outbreaks would be investigated per large outbreak.

### Clinical Isolates Clustering with Isolates of Food/Animal Origin

A total of 75 clusters contained isolates of both clinical and food/animal origin (Table). Seventeen clusters contained >5 clinical isolates, but only 1 clinical isolate matched the source isolate(s) in 34 clusters.

**Table:** Genetic clusters of *Campylobacter* isolates with matches between clinical isolates and food/animal isolates, Denmark, 2015–2017

Possible source	No. clusters with clinical isolates (1, 2–4, >5 isolates)	Total no. clinical isolates in clusters	Total no. cluster isolates
Domestic cattle	8 (5, 2, 1)	16	24
Imported chicken meat	10 (5, 5, 0)	18	30
Domestic chicken/broiler	55 (24, 16, 15)	192	322
Chicken meat*	13 (4, 4, 5)	69	89
Broiler	21 (14, 6, 1)	37	62
Chicken meat and/or broiler	21 (6, 6, 9)	86	171
Dog (domestic)	1 (0, 1, 0)	2	3
Chicken (imported) + turkey (imported) + broiler (domestic)	1 (0, 0, 1)	5	10
Total	75 (34, 24, 17)	233	389

*One cluster included a *C. jejuni* isolate from a dog.

Clinical isolates most often (192/233) matched isolates from domestic chicken meat, broilers, or both, corresponding to 25% of all clinical isolates in the study. In 10 clusters, 18 (2%) clinical isolates matched imported chicken meat, and 8 clusters included isolates from cattle and comprised a total of 16 (2%) clinical isolates. One small cluster matched 2 clinical isolates to an isolate from a dog, and a single cluster included isolates from imported chicken, imported turkey, and a domestic broiler. The chicken meat/broiler isolates in the same cluster were often linked to the same slaughterhouse, and some originated from the same farm. However, it was also common for cluster isolates to originate from different farms, but because complete data were not available, we could not make a systematic assessment.

**Figure 4:**
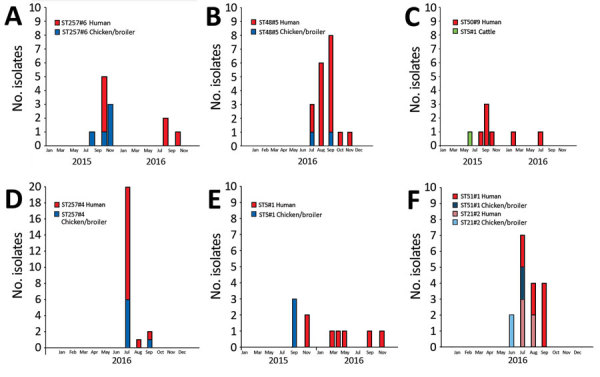
Epidemic curves for 6 large genetic clusters matching clinical *Campylobacter jejuni* isolates to source isolates, Denmark, 2015–2017. Each example shows the number of isolates (vertical) distributed over time (horizontal). All clusters include isolates that are within 4 allelic differences.  A) Cluster ST257#6, with 7 human cases and 2 peaks detected roughly a year apart. Patient cases primarily originated from 1 region (clinical microbiological laboratory [CML] Hvidovre, n = 6). The 5 domestic chicken/broiler isolates originated from 2 slaughterhouses, and 3 of these originated from different farms. B) ST48#5, the largest of all detected clusters, with 17 human cases, provided by all 4 CMLs. The cluster contained 2 domestic chicken isolates; however, no information about origin (i.e., slaughterhouse or farm) was available. C) ST50#9, the largest cluster detected with cattle as the potential source. A total of 7 domestically acquired clinical isolates were detected from August 2015 through July 2016; isolates were provided by all 4 CMLs. D) ST257#4, the second-largest cluster, comprising isolates from 16 human cases and 7 domestic chicken/broiler isolates, primarily (6/7) from the same slaughterhouse but from different farms, except for 2 isolates that were sampled 2 days apart. Clinical isolates were obtained from all 4 CMLs, primarily from Aalborg (n = 10). Thirteen infections were registered as domestically acquired; 2 patients had traveled and travel status was unknown for 1. E) ST5#1 genetic cluster with 7 patient cases, all domestically acquired; isolates received from 2 CMLs. The 3 domestic chicken/broiler isolates were obtained at the same slaughterhouse over 3 days; however, they originated from 2 farms only ≈20 km apart. F) ST51#1 and ST21#2, the 2 genetic clusters, each contains an isolate from patient A. Separately, the clusters comprise 8 and 5 isolates, respectively; combined, they cover 12 patients with domestically acquired infections representing all 4 CMLs. Of the 4 chicken/broiler isolates (domestic), 2 were obtained at the same slaughterhouse but originated from 2 farms with the same owner. No information was available for the other 2 isolates. ST, sequence type.

Clusters often originated with the detection of a food/animal isolate, followed by the presence of several clinical isolates over a few months, as seen in the examples of epidemic curves of large genetic clusters (Figure 4). In some instances, the cluster type disappeared for months and later reappeared in new patients (Figure 4, panels A, C, E). With few exceptions, the clinical isolates present in clusters were detected across the 4 geographic regions.

### Timeline of the Clusters

**Figure 5:**
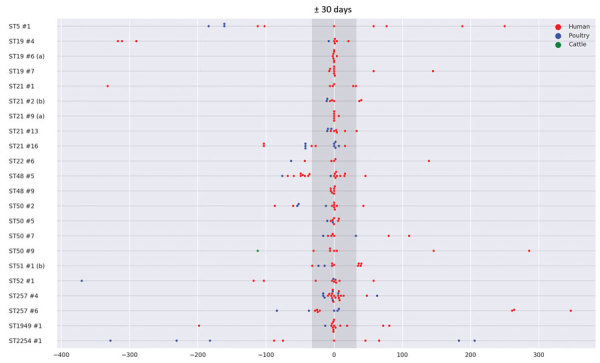
Distribution of large cluster isolates of *Campylobacter jejuni* from Denmark, over time. The 22 large genetic clusters (>5 clinical isolates) are listed vertically, and the cluster isolates are displayed over time (in days) horizontally. Each cluster is centered at the median date of the cluster isolates (t = 0). Each dot represents an isolate at a certain time, and colors indicate the origin of the isolate. The 6 clusters ST257#6, ST48#5, ST50#9, ST257#4, ST5#1, and ST51#1+ST21#2 are also illustrated in Figure 3. (a) indicates the 2 clusters representing the multistrain outbreak from the fifth CML; (b) indicates the 2 clusters linked by the same patient having an isolate part of each cluster.

Clusters occurred independently and generally reflected the seasonality of *Campylobacter* infections (Figure 2). Most clusters typically occurred over a few months. We determined the distribution of cluster isolates in the 22 large clusters over time (Figure 5). For most clusters, the isolates were distributed close in time around the median day (t = 0) of the specific cluster. Typically, most isolates were present within a 1-month period (+30 days) before and after this time point. However, it was not uncommon to detect a few cluster isolates outside this time range, and on a few occasions clusters lasted for several months (i.e., ST5#1 spanning 14 months and ST2254#1 spanning 17 months). 

### Potential Multistrain Outbreaks

In the dataset, we found 2 examples for which distinct genetic clusters could be linked by epidemiologic data. The first example was an identified outbreak in which 14 isolates were sequenced as part of an outbreak investigation. These isolates split into 2 distinct clusters (ST21#9 and ST19#6), consisting of 8 and 6 clinical isolates, respectively. This multistrain outbreak involved ≈100 schoolchildren who had been served unpasteurized milk while visiting a dairy farm.

In the second example, a sample from a patient was positive for 2 strains belonging to distinct clusters (ST51#1 and ST21#2; Figure 4, panel F). The 2 clusters were observed for the same period of 2016, and each matched isolates from domestic chicken meat and broilers within a few weeks and contained clinical isolates from the 4 regions. The 2 isolates from broilers representing each of the clusters were obtained from the same slaughterhouse; the broilers originated from 2 geographically close farms.

### Clusters of Food/Animal Isolates

Of the 66 clusters containing isolates of nonhuman origin, most (n = 59) included isolates from only 1 common source: domestic chicken meat and/or broilers (n = 37), domestic cattle (n = 11), imported chicken meat (n = 7), imported duck meat (n = 1), domestic pig (n = 1), imported turkey meat (n = 1), and domestic vegetables (n = 1). Isolates within each cluster often originated from the same farm, had been through the same slaughterhouse, or came from meat imported from the same country (data not shown). Cluster isolates were often isolated close in time, typically within a few days or months. Seven clusters contained isolates from >1 source: cattle and domestic chicken/broilers (3 clusters), cattle and dog (1 cluster), domestic chicken/broilers and dog(s) (2 clusters), and imported chicken and turkey meat and domestic chicken meat (1 cluster).

## Discussion

Our findings show that a large proportion of *Campylobacter* infections are not sporadic. We matched clinical isolates to genetic clusters (i.e., possible outbreaks), comprising almost half of the sequences from human infections during the study period. Given that only ≈10% of the diagnosed *Campylobacter* infections in Denmark were included in the study, we assume that if the remaining infections had been included, the proportion of sporadic cases would have decreased and the cluster sizes would have increased. A large proportion (30%) of all clinical isolates matched contemporary isolates from potential sources. In our WGS-based analysis of 1,509 isolates, a large fraction of the clinical isolates matched isolates from domestic broilers/chicken meat (25%) or imported chicken meat (2%), confirming that chicken is a main source of human infections.

Despite a large number of patient clusters (104), most contained few isolates and only 22 clusters harbored >5 isolates. Most (17/22) of the large clusters matched source isolates, primarily from domestic chicken meat and broilers. Only 5 large clusters did not match a source isolate. However, 3 of the 5 clusters represented solved outbreaks without an available source isolate, specifically the multistrain outbreak associated with raw milk (which encompassed 2 clusters) and a geographically confined outbreak for which the suspected source was lettuce (*6*). Thus, only 2 of the 22 large clusters were truly not matched to a source. In contrast, among the small clusters (2–4 clinical isolates), only a minority (29%) matched a source isolate. This finding indicates that domestic chicken meat and broilers are not dominant sources of small clusters and suggests that small clusters may arise from imported food, food with a lower contamination load, small batches of less widely distributed food, or nonfood sources such as direct animal contact or environmental exposures. This indication is supported by the fact that patient matches to sources other than domestic chicken meat/broilers (i.e., cattle, imported chicken, and dogs) were found primarily for clinical isolates in small clusters or isolates from sporadic cases. Because representative isolates from low-prevalence sources are difficult to obtain, we did not sufficiently cover these sources in this study.

The *C. jejuni* genomes in the study showed a high level of genetic diversity, in line with the general knowledge of the *C. jejuni* population structure, which is formed by a high level of horizontal gene transfer (*28*). Clearly, some clones are stable enough to be detected in patients and animals over several months, as indicated by our finding isolates with the same cluster type up to 17 months apart (Figure 5). The high diversity and presence of stable clones were difficult to deduce by previous typing methods. In recent years, WGS-based typing has proven to be a strong tool for detecting outbreaks caused by several bacterial pathogens; however, interpretation is not yet standardized. For cgMLST, the allelic distance that delimits outbreaks may vary among organisms, serotypes, and outbreak etiology and epidemiology. Thus, for most organisms, including *Campylobacter*, a defined cutoff is not established, although a cluster definition of 7 allelic differences has been proposed for *Listeria* outbreaks (*29*). Also for *Listeria*, however, several outbreaks with larger isolate diversity have been described (*30*). In this study, we used a cutoff of 4 allelic differences with UPGMA clustering after evaluating the phylogeny and defining clusters by using the available epidemiologic data (i.e., defined outbreaks and food/animal isolates obtained from the same location) (Appendix). UPGMA clustering limited the merging of closely related clusters seen with single-linkage clustering. The selected cutoff correctly matched the epidemiologically confirmed outbreaks and often clustered with chickens/broiler isolates from the same slaughterhouse/farm while separating isolates without apparent epidemiologic association. For future WGS-based analyses of *C. jejuni* for surveillance and outbreak detection, the available epidemiologic information needs to be considered to ensure correct inclusion and exclusion of isolates with respect to outbreak duration, possible source differences, and differences in genomic stability of the specific clone. Although it is not likely that a definite cutoff can be established, a cutoff of 4 allelic differences, or slightly more, seems to provide a sensible differentiation in a dataset like this one with limited a priori knowledge of the epidemiologic associations. However, UPGMA clustering is not useful in a real-time surveillance setup in which isolates are continuously added to the analysis. In routine surveillance, single-linkage clustering can be used by ensuring that newly added isolates are within a reasonable distance from the main cluster isolates because this method expands clusters and will sometimes join previously separated clusters.

This study shows that WGS is a valuable tool for improved surveillance and outbreak detection of *Campylobacter*, but a limitation is the detection of multistrain outbreaks (*31*). Multistrain outbreaks of *C. jejuni* are common, potentially constituting as much as 50% of reported outbreaks in the United Kingdom (*32*). Furthermore, food products, including chicken meat, may carry several types of *C. jejuni* (*33*). Thus, several clusters may be part of the same outbreak without being detectable in the current setup. For instance, the epidemiologically confirmed multistrain outbreak described in this study, which constituted 2 distinct clusters, would never have been connected without the epidemiologic link. Likewise, the single patient harboring strains from 2 genetic clusters would not have been detected systematically. Establishing the prevalence of multistrain outbreaks in the context of Denmark would be valuable, as would a cost-effective approach for detecting multistrain outbreaks in a routine surveillance setting.

Undertaking a detailed investigation of all clusters would be difficult and costly because many are small and many occur simultaneously. A carefully selected sampling frequency and investigation threshold facilitate a more cost-effective cluster investigation. With the aim of detecting large clusters, our study suggests that a sampling frequency of 10% is sufficient, although a low sampling frequency limits the value of epidemiologic investigations from food exposure data. In our study, most clusters had source links to chicken, a commonly consumed food source, making exposure investigation less efficient. Domestically produced chicken meat is distributed throughout the country, which is reflected by patient cases in clusters generally not being confined to a single geographic location, often limiting the geographic signal. Although the principal part of the clusters is confined to a short period, the general picture of clusters lasting for a few months offers the potential to prevent some cases through traditional outbreak detection and follow-up. This study indicates that it might be possible to limit some large clusters and probably a substantial part of sporadic cases by using control measures in the poultry industry. However, it is more difficult to identify other sources (e.g., raw milk, vegetables, animal contact, and different kinds of environmental exposures) because doing so would take disproportionate resources to encompass sufficient routine sampling of these sources. Because food/animal isolates are widely distributed in the phylogenetic tree, our study indicates that we cannot expect to be able to predict the source of human infections without having isolates from the specific source. In addition, the *C. jejuni* clones causing the 2 point-source outbreaks with a probable nonpoultry source (i.e., raw milk and lettuce) were also genetically similar, albeit not considered matches, to strains isolated from poultry.

This study confirms previous findings that campylobacteriosis is not a wholly sporadic disease but rather constitutes numerous small clusters with cases geographically distributed across the country, lasting for a few months and then being replaced by other clusters of different strains of *C. jejuni*. As a result of this study, continuous surveillance based on sampling of ≈10% of the patient cases has been initiated in Denmark for the purpose of detecting large outbreaks and linking them to contemporary food/animal sources to improve public health and reduce the incidence of *Campylobacter* infections.

Appendix Cost-effectiveness calculation and phylogenies of the 2 point-source outbreaks and a segment of the isolates in study of use of whole-genome sequencing to detect *Campylobacter jejuni* outbreaks and match patient isolates to sources, Denmark, 2015–2017.
