# Supplementary material for: Whole-Genome Sequencing to Detect Numerous Campylobacter jejuni Outbreaks and Match Patient Isolates to Sources, Denmark, 2015–2017
Source: Emerg Infect Dis. 2020 Mar;26(3):523–32. doi: 10.3201/eid2603.190947 (PMC7045838; doi:10.3201/eid2603.190947)
Supplement: Appendix — Cost-effectiveness calculation and phylogenies of the 2 point-source outbreaks and a segment of the isolates in study of use of whole-genome sequencing to detect Campylobacter jejuni outbreaks and match patient isolates to sources, Denmark, 2015–2017. [file 19-0947-Techapp-s1.pdf]

Article DOI: https://doi.org/10.3201/eid2603.190947

Whole-Genome Sequencing to Detect Numerous *Campylobacter jejuni* Outbreaks and Match Patient Isolates to Sources, Denmark, 2015–2017

Appendix

Cost-Effectiveness Calculation

```
#!/usr/bin/env python3

from scipy.stats import binom

"""
How should the scenarios work

Small outbreaks are noise and will be a waste of time if investigated.
Large outbreaks are significant and must be found with at least 95%
probability

"""
##Presumed constants
n_small = 10   # small outbreaks with total size
n_large = 100  # large outbreaks with total size
n_early = 33   # Early detection defined as within this number of
cases
f_inv = 101    # Inverse frequency of large outbreaks

##Variabler
p = 0.10 # sampling frequency
x = 3 # threshold for investigation -1

## Probability of observing a large outbreak
p_large = binom.sf(x,n_large,p)
print("p_large = {}".format(p_large))

p_early = binom.sf(x,n_early,p)
print("p_early = {}".format(p_early))

## Probability of observing a single small outbreak
p_small = binom.sf(x,n_small,p)
print("p_small = {}".format(p_small))
```

```
## Mean number of small outbreaks observed
mean = f_inv*p_small
print("Number of small outbreaks observed pr large outbreak
{}".format(mean))

## Frequency of small outbreaks to large
f_small = mean/p_large
print("Number of small outbreaks observed pr large outbreak observed
{}".format(f_small))
```

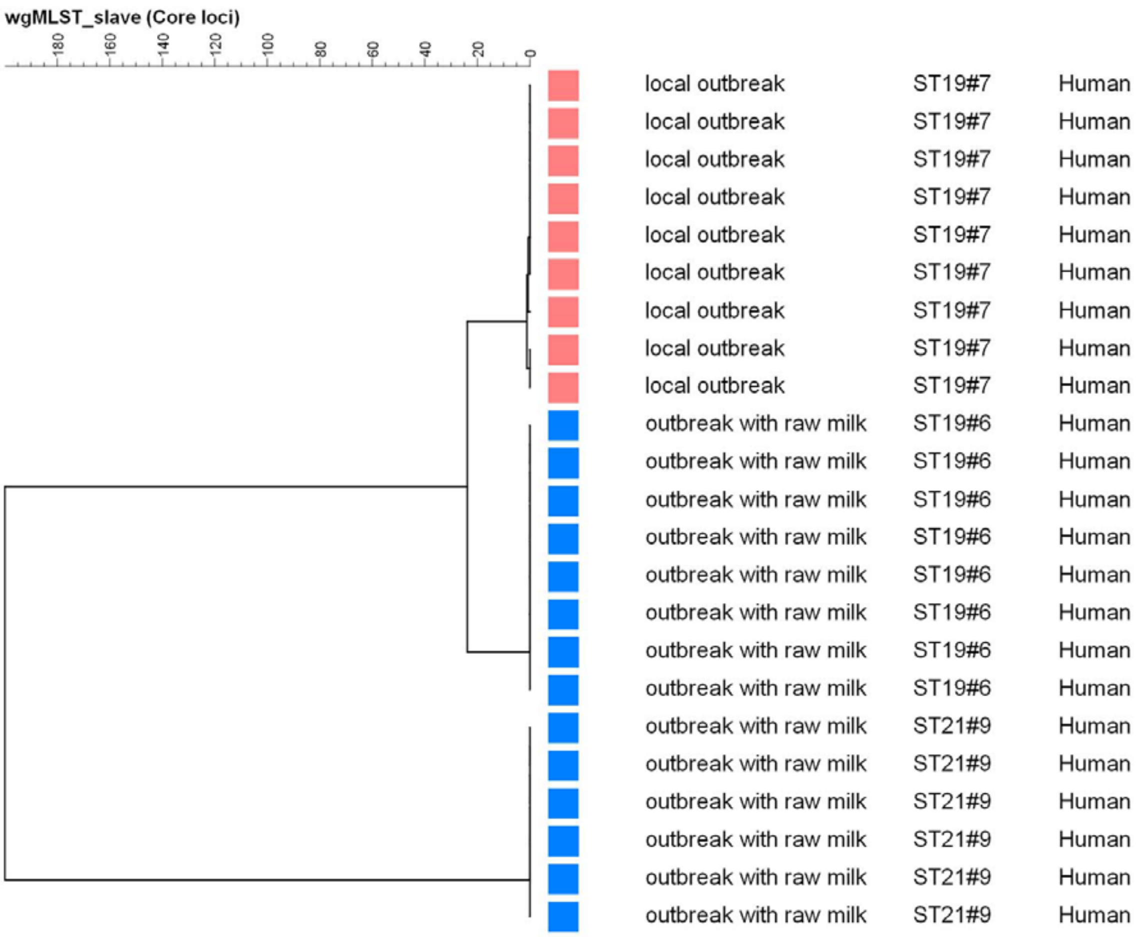

Appendix Figure 1. Phylogeny of the 2 point-source outbreaks in study of use of whole-genome sequencing to detect *Campylobacter jejuni* outbreaks and match patient isolates to sources, Denmark, 2015–2017.

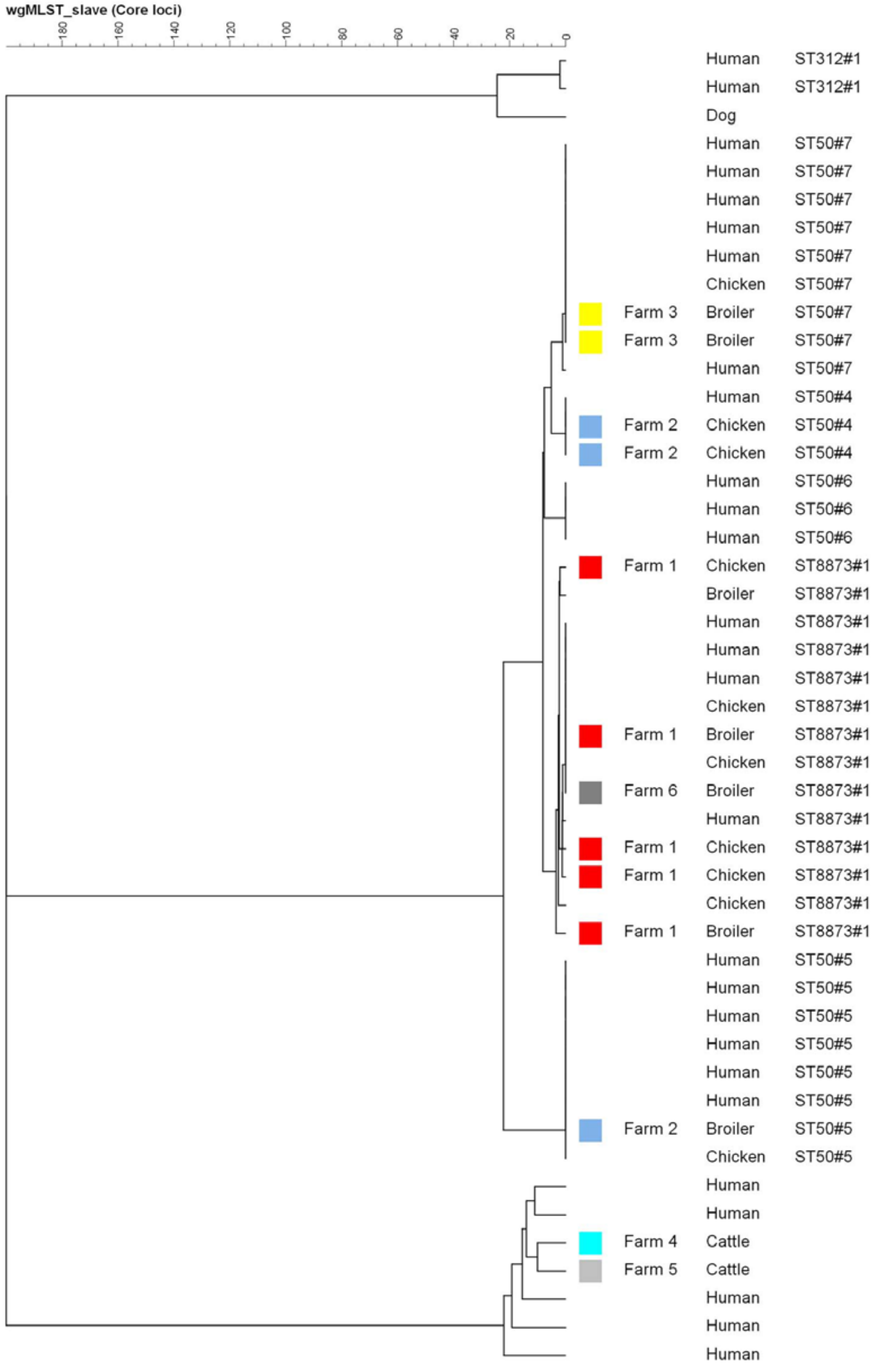

Appendix Figure 2. Phylogeny of a segment of the isolates in study of use of whole-genome sequencing to detect *Campylobacter jejuni* outbreaks and match patient isolates to sources, Denmark, 2015–2017.
